# MicroRNA: Crucial modulator in purinergic signalling involved diseases

**DOI:** 10.1007/s11302-022-09840-y

**Published:** 2022-02-02

**Authors:** Jing Guo, Peng Yang, Yi-Fan Li, Jin-Fan Tang, Zhao-Xuan He, Shu-Guang Yu, Hai-Yan Yin

**Affiliations:** School of Acupuncture and Tuina, Chengdu University of Traditional Chinese Medicine, Chengdu, 610075 China

**Keywords:** Purinergic signalling, microRNAs, Cancers, Gastrointestinal diseases, Cardiovascular diseases, Neurological diseases, Ophthalmic diseases, Musculoskeletal diseases

## Abstract

Both microRNAs (miRNAs) and purinergic signalling are widely and respectively expressed in various tissues of different organisms and play vital roles in a variety of physiological and pathological processes. Here, we reviewed the current publications contributed to the relationship of miRNAs and purinergic signalling in cardiovascular diseases, gastrointestinal diseases, neurological diseases, and ophthalmic diseases. We tried to decode the miRNAs-purinergic signalling network of purinergic signalling involved diseases. The evidence indicated that more than 30 miRNAs (miR-22, miR-30, miR-146, miR-150, miR-155, miR-187, etc.) directly or indirectly modulate P1 receptors (A_1_, A_2A_, A_2B_, A_3_), P2 receptors (P2X1, P2X3, P2X4, P2X7, P2Y2, P2Y6, P2Y12), and ecto-enzymes (CD39, CD73, ADA2); P2X7 and CD73 could be modulated by multiple miRNAs (P2X7: miR-21, miR-22, miR-30, miR-135a, miR-150, miR-186, miR-187, miR-216b; CD73: miR-141, miR-101, miR-193b, miR-340, miR-187, miR-30, miR-422a); miR-187 would be the common miRNA to modulate P2X7 and CD73.

## Introduction

microRNAs (miRNAs) are ~ 22 nucleotides (nt) sequences of small RNA regulating gene expression at the posttranscriptional level [1]. miRNAs modulate the target mRNA by matching with its complementary sequences in the 3′-UTR of target mRNA, thus leading to mRNA silencing, degradation, or translation repression [2, 3]. To date, it has been identified that miRNAs are expressed in almost all studied multicellular eukaryotes in the plant and animal kingdoms and even encoded by certain single-cell eukaryotes and by viruses. In humans, more than 60% of protein-coding genes contain at least one conserved miRNA binding site [4]. A growing number of evidence revealed that miRNAs are powerful regulators of various cellular activities, including cell growth, proliferation, differentiation, development, homeostasis, and apoptosis [5]. They are actively involved in various pathological conditions, such as cancer, diabetes mellitus, Alzheimer’s disease, Parkinson’s disease (PD), cardiovascular disease, and metabolic disease [2, 6]. Moreover, the use of miRNA-based therapeutics has dual advantages. First, miRNAs are naturally occurring molecules in human cells, unlike man-made chemotherapy compounds or antisense oligonucleotides (ASOs), and therefore have all the mechanisms in place for their processing and downstream target selection. Second, miRNAs act by targeting multiple genes within one pathway, thus causing a broader yet specific response. miRNAs have been considered as a novel type of biomarkers and potential therapeutic targets for various diseases.

Purinergic signalling is referred to some purines (ATP, ADP, UDP, UTP, adenosine, etc.) acting as endogenous ligands that bind to and activate plasmalemmal purinergic receptors (P1 receptors and P2 receptors), which mediate extracellular communication [7]. In addition, some associated ecto-enzymes, such as CD39, CD73, and adenosine deaminase (ADA), also get involved in Purinergic signalling [8, 9]. P1 receptors are G protein-coupled receptors (GPCRs) that recognize adenosine as endogenous ligand and are divided into four subtypes known as A_1_, A_2A_, A_2B_, and A_3_ receptors. P2 receptors are consist of two subgroups: P2X (ATP-gated ion channels: P2X1-7) and P2Y (metabotropic GPCRs: P2Y1, P2Y2, P2Y4, P2Y6, P2Y11, P2Y12, P2Y13, and P2Y14) receptors [10]. P2Y receptors are activated by ATP, UTP, ADP, and UDP, whereas P2X is only activated by ATP. Purinergic receptors are expressed by almost every cell type, even in very primitive organisms, phylogenetically [11]. They have been implicated in embryogenesis, organogenesis, postnatal development, and aging in vertebrates [12–14]. Purinergic signalling is cross-linked with other transmitter networks to coordinate numerous aspects of cell behavior such as proliferation, differentiation, migration, apoptosis, and other physiological processes critical for the proper function of organisms. Pathological deregulation and malfunction of purinergic signalling contribute to the pathology of numerous diseases, including gout, diabetes, depression, hypertension, heart failure, neurodegeneration, rheumatic immune diseases, inflammation, chronic pain, and cancer [7, 15]. Accordingly, purinergic signalling is also considered as a potential therapeutic target for numerous diseases.

**Fig. 1:**
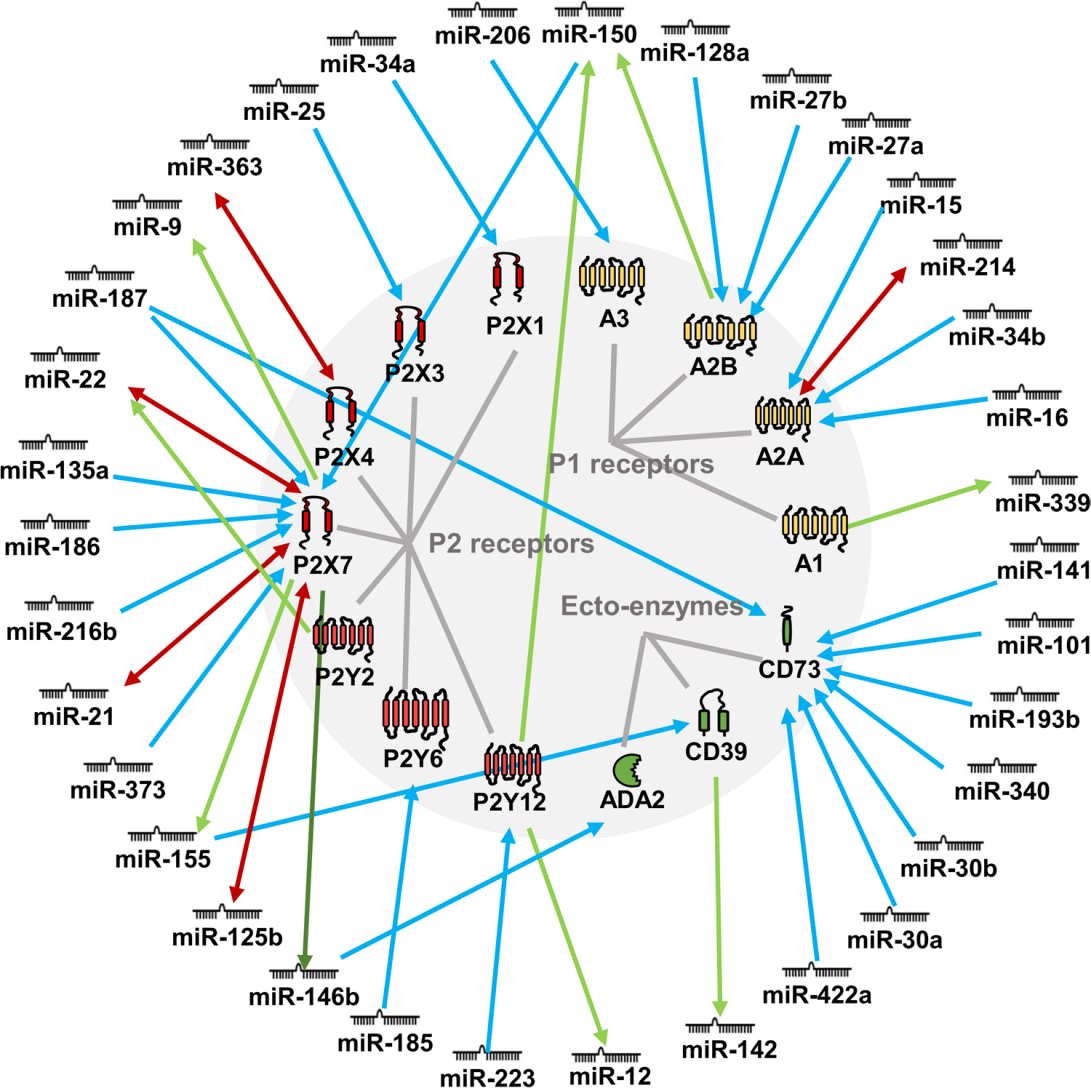
Interaction between purinergic signalling and miRNAs. The whole P1 receptor family (A_1_, A_2A_, A_2B_, A_3_) are correlated with miRNAs. Among the P2 receptor family, P2X1, P2X3, P2X4, P2X7, P2Y2, P2Y6, and P2Y12 were currently found to correlate with miRNAs. CD39, CD73, and ADA2 are modulated by miRNAs, as well. Typically, purinergic signalling is posttranscriptionally modulated by miRNAs (blue arrow). Intriguingly, some purinergic signalling modulates miRNAs in turn (green arrow), while part of them can modulate each other mutually (red two-way arrow). Apparently, miR-187 modulates multiple purinergic signalling, P2X7 receptors and CD73. A_2A_, A_2B_, P2X7 receptors, and CD73 are modulated by multiple miRNAs

In view of the fact that both miRNAs and purinergic signalling are widely and respectively expressed in various tissues of different organisms, playing vital roles in physiological and pathological processes, the question is raised: Do and how do miRNAs modulate purinergic signalling? In recent years, increasing evidence on miRNAs and purinergic signalling has been released, which might provide some clues for answering the question. Therefore, we reviewed the relative experimental articles that contributed to the function of miRNAs in purinergic signalling involved diseases (Table 1 and Fig. 1), tried to explore the miRNAs-purinergic signalling network in diseases, and provide a deeper understanding of this research field.

**Table 1:** List of purinergic receptors correlated with miRNAs in diseases

Purinergic receptors		Regulation*	miRNAs	Directly targeted?^#^	Concerned disease/model	Year	Refs
P1	A_1_	→	miR-339	-	Spontaneously hypertensive	2014	[116]
	A_2A_	←	miR-16	√	Ulcerative colitis	2016	[44]
		←	miR-15, miR-16, miR-214	√	Inflammation	2012	[107]
		← →	miR-214	√	Inflammation	2015	[106]
		←	miR-34b	√	Parkinson’s disease	2014	[70]
	A_2B_	←	miR-27b, miR-128a	√	Colitis	2010	[46]
		←	miR-27a	-	Bronchiolitis obliterans organizing pneumonia (BOOP)	2017	[114]
		→	miR-150	-	Myocardial infarction	2013	[117]
	A_3_	←	miR-206	√	Ulcerative colitis	2017	[47]
P2	P2X1	←	miR-34a	-	Overactive bladder (OAB)	2012	[115]
	P2X3	←	miR-25	-	Overactive bladder (OAB)	2012	[115]
	P2X4	← →	miR-363-5p	√	Nerve injury	2021	[64]
	P2X7	→	miR-9	-	Painful diabetic neuropathy (PDN)	2017	[118]
		←	miR-21	-	Non-small cell lung cancer (NSCLC)	2015	[19]
		→	miR-21	-	Psoriasis	2013	[119]
		←	miR-22	-	Epilepsy	2015	[78]
		→	miR-22, miR-155, miR-125b, miR-146b	-	Amyotrophic lateral sclerosis (ALS)	2013	[120]
		←	miR-125b	-	Amyotrophic lateral sclerosis (ALS)	2016	[81]
		←	miR-135a	√	Traumatic spinal cord injury (SCI)	2019	[68]
		←	miR-150	√	Breast cancer	2013	[17]
		←	miR-150	√	Intervertebral disc degeneration (IDD)	2020	[102]
		←	miR-150	√	Myocardial infarction (MI)	2015	[58]
		←	miR-150, miR-186	√	Cervical cancer	2008	[16]
		←	miR-187-3p	√	Ischemia reperfusion (IR)	2019	[84]
		←	miR-187	√	Chronic ocular hypertension	2018	[92]
		←	miR-216b	√	Breast cancer	2014	[18]
		←	miR-373	-	Osteoarthritis	2018	[97]
		←	miR-373	√	Osteoarthritis	2017	[98]
	P2Y2	→	miR-22	-	Inflammation	2015	[121]
	P2Y6	←	miR-185	√	Hypertension	2017	[57]
	P2Y12	→	miR-12, miR-150	-	Coronary artery disease	2020	[122]
		←	miR-223	√	Stroke and acute coronary syndromes	2009	[60]
Enzymes	CD39	→	miR-142-3p	-	Vascular inflammation	2018	[123]
		←	miR-155	-	Sepsis	2015	[109]
		←	miR-155	-	Allergic airway inflammation (AAI)	2015	[112]
	CD73	←	miR-30a	√	Colorectal cancer (CRC)	2017	[21]
		←	miR-30a-5p	√	Non-small cell lung cancer (NSCLC)	2017	[23]
		←	miR-30b, miR-340	√	Gallbladder carcinoma	2018	[24]
		←	miR-101-3p, miR-141-3p, miR-340-5p	-	Breast cancer	2021	[26]
		←	miR-187	√	Colorectal cancer (CRC)	2016	[22]
		←	miR-193b	√	Pancreatic cancer	2012	[25]
		←	miR-422a	-	Head and neck squamous cell carcinoma (HNSCC)	2016	[20]
	ADA2	←	miR-146b-3p	√	Diabetic retinopathy (DR)	2015	[94]
		←	miR-146b-3p	√	Diabetic retinopathy (DR)	2017	[95]

^*^ ← purinergic signalling was modulated by miRNAs, → purinergic signalling modulates miRNAs in turn.^#^√purinergic signalling was directly targeted by miRNAs, and it was confirmed by luciferase reporter assay

## Purinergic signalling modulated by miRNAs in cancers

It has been known that both purinergic signalling (ATP, adenosine, P1 and P2 receptors) and numerous miRNAs have close relationship to cancer. For example, tumor microenvironment is rich in adenosine and ATP, and several types of P2 receptors are involved in the suppression of tumor growth, including P2X5, P2X7, P2Y1, P2Y2, and P2Y11 receptors. Nowadays, increasing evidences were reported that miRNAs take part in the purinergic signalling modulation in many cancers, such as cervical cancer, breast cancer, and non-small cell lung cancer (NSCLC), head and neck squamous cell carcinoma (HNSCC), colorectal cancer (CRC), gallbladder carcinoma (GBC), and pancreatic cancer. Currently, purinergic P2X7 receptor and the enzyme CD73 were mainly found to be modulated by different miRNAs in a set of cancer. To be specific, P2X7 receptors were verified to be modulated by miRNAs in cervical cancer, breast cancer, and NSCLC. CD73 was modulated by miRNAs in HNSCC, CRC, NSCLC, GBC, and pancreatic cancer (Table 1).

In cancer epithelial cells from cervical cancer patients, increased expression of miR-186 and miR-150 was detected. And both miR-186 and miR-150 were found to decrease P2X7 mRNA by activation of instability sites of the two miRNAs located at the 3′-UTR-P2X7. Besides, the combination of the mimic or inhibitors of the two miRNAs showed a more significant effect than a single drug, respectively [16]. In the like manner, miR-150 was found overexpressed in breast carcinoma tissues and cell lines. In breast cancer cell lines, blocking the action of miR-150 with inhibitors leads to cell death, while ectopic expression of the miR-150 results in increased cell proliferation [17]. However, miR-216b was found downmodulated, while P2X7 receptors were upmodulated in breast cancer. Ectopic expression of miR-216b mimics leads to inhibited cell growth and apoptosis, while blocking the expression of miR-216b results in increased cell proliferation [18]. Conversely, it was observed miR-21 in NSCLC patients with K-Ras mutations showed a high level, and it corresponded to low expression of P2X7 receptors and decreased survival of NSCLC patients [19].

The evidence of CD73 modulated by miRNAs in cancers has been reported in HNSCC, CRC, NSCLC, GBC, pancreatic cancer, and breast cancer. In HNSCC, a significant negative correlation between expression levels of miR-422a and CD73 mRNA was found [20]. In CRC, miR-30a was found to play an important role in regulating cell proliferation and apoptosis and thus affecting the growth of the tumor by regulating CD73 [21]. Another study showed that miR-187 expression was downmodulated in CRC tissues, and it was able to directly suppress the expression of several essential upstream effectors of Smad pathway, including CD73 [22]. In NSCLC, direct regulation of CD73 through miR-30a-5p was described. CD73 was overexpressed while miR-30a-5p was underexpressed in NSCLC tissues compared with adjacent noncancerous, and in NSCLC cell lines, overexpression of miR-30a-5p inhibited cell proliferation in vitro and in vivo [23]. The EGF signalling pathway was involved in the process above. In GBC, miR-30b, another member of the miR-30 family, was shown to modulate CD73. In the same study, miR-340 was verified to have a similar function with miR-30b in reducing GBC cell proliferation, migration, and invasion when overexpressed [24]. In pancreatic cancer, miRNAs involved in the mitogen-activated protein kinase (MAPK) were noticed. And a search for target genes of miR-193b led to the identification of 6 different factors, including CD73, but effects of miR-193b overexpression on CD73 expression on mRNA and protein level were not analyzed [25]. In breast cancer, several breast cell lines were utilized to determine the gene expression of CD39 and CD73, and it was confirmed in silico analyses that the miRNAs miR-101-3p, miR-141-3p, and miR-340-5p were highly expressed in MCF7 cells and targeted CD73 [26].

Considering purinergic signalling modulated by miRNAs in cancer, the importance of the P2X7 receptor and CD73 has been proved. P2X7 receptor has been shown to play key roles in the development and spread of tumor cells because the P2X7 receptor could either drive cell survival and proliferation or induce cell death, depending on its activation state [27]. Uterine epithelial cells in humans showed that baseline and the P2X7 receptor-mediated apoptosis are lower in cancer cells than in normal cells [28–30]. And it was reported to be modulated by miR-186, miR-150, miR-216b, and miR-21 in diverse cancers. As a major enzymatic source of extracellular adenosine in the purinergic signalling system, CD73 hydrolyzes AMP to adenosine which is able to activate the four adenosine receptors: A_1_, A_2A_, A_2B_, A_3_ [9]. Beyond that, CD73 is a signal and adhesive molecule that modulates cell interaction with extracellular matrix components, so that plays a pivotal role in mediating the invasive and metastatic properties of cancers [31–33]. Anti-CD73 therapy is a promising approach for cancer treatment in the future [23, 34, 35]. It was indicated that the P2X7 receptor and CD73 are promising therapeutic targets. miR-150, a hematopoietic cell-specific miRNA, was shown to affect B-cell differentiation and development [36]. Different studies have suggested that miR-150 is significantly overexpressed in multiple kinds of cancers, including malignant lymphoma, gastric, lung, endometrial, and pancreatic cancers [16, 37–39]. According to the present evidence, miR-150 could modulate P2X7 receptors in cervical cancer and breast cancer. It indicates that miR-150 might be a crucial target in treating purinergic involved cancer. Besides P2X7 and CD73, if other purinergic signalling related to cancer also could be modulated by miRNAs? It still needs to be further explored. In breast cancer, it was reported that P2X7 has a dual function in some cancer, for example, when activated for a short time, P2X7 promotes metastasis, but when activated for long periods, it is related to breast cancer cell death [40]. The dual function of P2X7 in different stages of tumor is led by fine-tuning regulation of multiple miRNAs. It was known that miR-150 and miR-187, and maybe some other unknown miRNAs targeting P2X7, play important role in breast cancer.

## Purinergic signalling modulated by miRNAs in gastrointestinal diseases

Among gastrointestinal diseases, the regulation of miRNAs to P1 (A_2A_, A_2B_, A_3_) receptors was supported by diverse evidences. In ulcerative colitis (UC) patients, the expression of miR-126, miR-150, and miR-155 was significantly upmodulated. These miRNAs contribute to the inflammatory reaction via regulating the nuclear factor-kappa B (NF-κB) signalling pathway [41–43]. Similarly, the expression of miR-16 in UC patients was found increased, so that influenced the activation of NF-κB signalling pathway as well as proinflammatory cytokine production. This influence was identified to accomplish via the mediation of A_2A_ receptors. And the A_2A_ receptor is a direct target of miR-16. Inhibitions of miR-16 might help normalize the upregulated A_2A_ receptors in UC patients [44]. A_2B_ receptors were modulated by miRNAs, as well. In both humans and models of inflammatory bowel disease, A_2B_ receptors mRNA and protein expression were increased and were demonstrated to be modulated by TNF-α [45]. Further research of the author revealed that TNF-α-induced A_2B_ receptors expression in colonic epithelial cells is posttranscriptionally and negatively modulated by miR-27b and miR-128a [46]. But combining the two relative studies, we shall notice that the miR-27b and miR-128a seem to be mediators in TNF-α inducing A_2B_ receptors expression [45, 46]. Unlike A_2B_ receptors, A_3_ receptor levels were significantly downmodulated, the expression of miR-206 was upregulated in UC patients; however, miR-206 was proved to have a proinflammatory role in UC by downregulating A_3_ receptors expression and activating NF-κB signalling [47]. A further study found that downregulation of miR-206 by long-term mesalamine treatment may confer a protective effect in inducing and maintaining histologic remission in UC [48].

According to the present evidence, the modulation of miRNAs to purinergic signalling was mainly reflected on A_2A_, A_2B_, and A_3_ receptors in colitis. It seems that miRNAs mainly play a proinflammatory role and negatively modulate A_2A_ receptors and A_3_ receptors via NF-κB signalling pathway in gastrointestinal diseases. In fact, the involvement of the A_1_ receptor in colitis has been reported [49]. But no evidence shows it was modulated by miRNAs in colitis or other gastrointestinal diseases yet. Besides, P2X and P2Y receptors are widely expressed in the gastrointestinal tract and participate in sympathetic transmission and neuromodulation involved in enteric reflex activities, as well as influencing gastric and intestinal epithelial secretion and vascular activities [50]. It was demonstrated that the pharmacological manipulation of purinergic signalling represents a viable way to counteract several gastrointestinal diseases [50]. For example, purinergic-mediated strategies as adjunctive treatments were used to correct immune dysregulation in inflammatory bowel disease (IBD) patients [51]. Whether the P2 receptor was modulated by miRNAs in gastrointestinal diseases remain to be studied.

## Purinergic signalling modulated by miRNAs in cardiovascular diseases

In the cardiovascular system, purinergic signalling was involved in the physiological and pathological processes, such as vascular tone regulation, cell damage, inflammation, and injury [52]. The potential of purinergic signalling in cardiovascular diseases has been noticed and discussed, including heart failure, infarction, angina, coronary artery disease, hypertension, and ischemia [53]. In cardiovascular disease, evidence illustrated the modulation of miRNAs on P2 receptors, including P2X7 receptors, P2Y6 receptors, and P2Y12 receptors.

miRNAs showed the potential in therapeutic application treating hypertension by modulating purinergic signalling. In cardiovascular disease, angiotensin II (Ang II) promotes vascular smooth muscle cell proliferation, hardens the vessel wall, and causes the lumen narrow [54]. Activation of the P2Y6 receptor, an inflammation-inducible G protein-coupled receptor, induces a Ca^2+^ response in vascular smooth muscle cells that results in contraction of isolated blood vessels [55]. It was revealed that P2Y6 receptors promote Ang II-induced hypertension [56]. A miR-185/P2Y6 axis was found to inhibit Ang II-induced human aortic vascular smooth muscle cells (HAVSMCs) proliferation through miR-185, negatively regulating the P2Y6 receptor expression and the downstream ERK pathway [57]. Rescuing miR-185 expression to inhibit P2Y6 may represent a therapeutic strategy against HAVSMC dysfunction and hypertension.

Through modulating purinergic signalling, miRNAs might protect the heart from ischemic injury. Genetic deletion of miR-150 in mice causes abnormalities in cardiac structural and functional remodeling after myocardial infarction (MI). And miR-150 was confirmed functioning as a protective miRNA in regulating cell death by repressing P2X7 receptors, as well as pro-apoptotic egr2, a zinc-binding transcription factor induced by ischemia [58]. Platelets play a critical role in cardiovascular disease involved in thrombosis and the incorrect function of which is strictly associated with stroke or MI [59]. P2Y12 receptors are known to amplify aggregation induced by all known platelet agonists. Human platelets harbor an abundant and diverse array of miRNAs, and it was ascertained the P2Y12 receptor expression might be subjected to miR-223 control in human platelets [60].

The role of miRNAs and purinergic signalling in cardiovascular diseases has been described from different aspects, such as cardiac structural and functional remodeling, blood pressure, etc. But only three purinergic receptors were involved. In fact, the therapeutic potential of purinergic signalling for vascular disease is clearer, and various antagonists and agonists are being considered [61]. More purinergic signalling might be modulated by miRNAs, though not known.

## Purinergic signalling modulated by miRNAs in neurological diseases

Among the seven members of the P2X family, the P2X7 receptor has been the most focused one in neurological diseases [62]. According to the present evidence, mainly the P2 receptors, including P2X4, P2X7, and P2Y12 receptors, were reported to be modulated by miRNAs in neurological diseases. A_2A_ receptors were also involved. To be specific, miR-34b modulates A_2A_ receptors, miR-363-5p modulates the P2X4 receptor, and P2X7 receptors are modulated by miR-135a, miR-22, and miR-187-3p. Moreover, the role of a purinergic signalling blocker and the indirect correlation between P2X7 and miR-15b were also reported.

The P2X4 and P2X7 receptors were reported to be modulated by miRNA in peripheral nerve injury and spinal cord injury, respectively. P2X4 receptors in nerves have been discovered contributing to the fate and survival of activated microglia [63]. The interaction of P2X4 and miRNA in Schwann cells, major cells of the peripheral nervous (PNS), which undergo biochemical and morphological alterations after nerve injury, was described. It was identified that miR-363-5p was elevated in rat sciatic nerves during the postnatal development period and decreased after nerve injury. Meanwhile, P2X4 receptors were negatively related with and directly targeted by miR-363-5p [64]. Interestingly, a P2X4 receptor antagonist increased the levels of miR-363-5p. In brief, the correlation between miR-363-5p and the P2X4 receptor contributes to the dedifferentiation and migration of Schwann cells after nerve injury [64]. In spinal cord injury (SCI), blockage of P2X7 receptors and other purinergic receptors provides neuroprotection in rats [65–67]. And a study holds that miR-135a is a potential therapeutic target for SCI because miR-135a downregulation causes overexpression of P2X7 receptors and increases excitotoxicity when exposed to the high concentration of ATP. Conversely, overexpression of miR-135a leads to reduced expression of the P2X7 receptor and an amelioration of the calcium response to excitotoxic levels of ATP [68].

The modulation of miRNAs to purinergic signalling was revealed in PD and epilepsy. An indirect correlation between miRNA and purinergic signalling was reported in amyotrophic lateral sclerosis (ALS). In PD, A_2A_ receptors play out antagonistic interaction with dopamine D_2_ receptors [69]. Increased striatal A_2A_ receptor levels were an early event in PD disease-related pathology, and it is directly targeted by miR-34b [70]. It was proposed that early deregulation of miR-34b/c in PD triggers downstream transcriptome alterations underlying mitochondrial dysfunction and oxidative stress, which ultimately compromise cell viability [71]. Therefore, new insights into the molecular mechanism underlying the progression of PD were provided, and miR-34b might be a potential therapeutic target in treating PD. P2X7 levels were increased in experimental models of epilepsy and in resected brain tissue from pharmacoresistant temporal lobe epilepsy (TLE) patients [72–74]. Pharmacologic blockage or genetic ablation of the P2X7 receptor reduced seizure severity during prolonged seizures (status epilepticus) in rodents [72, 75–77]. A role for posttranscriptional regulation of the P2X7 receptor has been reported, and it was suggested that therapeutic targeting of miR-22 may prevent inflammation and the development of a secondary epileptogenic focus in the brain [78]. The correlation of miRNAs and P2X7 receptors in ALS was also revealed, although not as direct as others. miR-125b was directly activated by NF-κB and shown to cause constitutive NF-κB activation by suppressing the expression of A20, a ubiquitin-editing enzyme also known as TNF alpha-induced protein 3 (TNFAIP3) [79, 80]. A further study established that the existence of a pathological circuit in which termination of A20 function by miR-125b strengthens and prolongs the noxious P2X7 receptor-dependent activation of NF-κB in microglia, with deleterious consequences on motor neurons [81].

Purinergic signalling was involved in ischemia reperfusion (IR) and able to alleviate pain. Hyperalgesia and allodynia have been suggested as significant challenge for almost all patients who have experienced ischemia reperfusion (IR) insults [82, 83]. miR-187-3p was revealed to play a role in alleviating pain induced by IR through inhibiting spinal P2X7 receptors [84]. In IR-injured mice, pain hypersensitivity was alleviated when treated with mimic-187-3p or the P2X7 receptor antagonist. While in sham-operated mice, pain hypersensitivity was induced when treated with inhibitor-187-3p or the P2X7 receptor agonist [84]. The role of the purinergic signalling blocker, prasugrel, was also concerned. Prasugrel was reported to upregulate the downregulated expression of miR-22 in IR and thus partook in preserving hippocampal cellularity [85]. Strictly speaking, what was verified in this study was the aptitude of the P2Y12 blocker, prasugrel, to overexpress miR-22 in an animal model of transient global cerebral ischemia. The correlation between P2Y12 itself and miR-22 needs to be further developed. In fact, the importance of miR-22 in neurological diseases has already been noticed. miR-22 might be a potential therapeutic target because it was reported to possess neuroprotective effects that nominate it to be used in treating cerebral IR injury [86, 87].

In summary, miRNAs were found to modulate purinergic signalling in nerve injury, including peripheral injury and spinal cord injury, central nervous system disease, including PD and epilepsy, and pain hypersensitivity induced by IR. In addition, the indirect correlation between miRNAs and purinergic signalling, role of purinergic signalling blocker, also implied the potential of miRNAs and purinergic signalling. From the peripheral to the central, direct targeting to indirect correlation, and purinergic signalling itself to pharmacology, studies concerning miRNAs and purinergic signalling in neurological diseases seem to be relatively thorough; the potential therapeutic application of miRNAs might happen in this field.

## Purinergic signalling modulated by miRNAs in ophthalmic diseases

The P2X7 receptor was distributed throughout all layers of the retina and associated with the death of photoreceptor and retinal ganglion cell (RGC) because of its activation by extracellular ATP[88]. And it has been identified to induce glaucomatous RGCs apoptosis by elevating intracellular Ca^2+^ [89, 90]. On the other hand, it was confirmed that miR-187 promoted RGCs survival, while decreased miR-187 induced RGCs apoptosis through upregulating Smad7 in vitro [91]. Indeed, miR-187 plays an essential role in retina tissue by correlating with P2X7 receptors in glaucoma. Through negatively regulating P2X7 receptors, miR-187 inhibits the oxidative stress-induced apoptosis of RGC-5 cells, and overexpression of miR-187 could alleviate oxidative stress injury in retina tissue of rat models with chronic ocular hypertension [92]. Therefore, miR-187 could positively regulate retinal cells survival through negatively regulating P2X7 receptors.

Interestingly, although ADA2 was not expressed in the rodent retina, it was suggested to be a key regulator of immune and inflammatory responses in the porcine and human retina [93]. In the vitreous of diabetic patients, decreased miR-146b-3p is associated with increased ADA2 activity. Ectopic expression of miR-146-3p suppressed ADA2 expression, activity, and TNF-α release in the AGA-treated human macrophages [94]. Subsequent research holds that ADA2 was implicated in the breakdown of the blood retinal barrier (BRB) in DR through macrophages-derived cytokines, while inhibition of ADA2 by miR-146-3p might be a valuable tool to preserve BRB in DR [95].

Briefly, P2X7 receptors were found to be modulated by miR-187 in glaucoma, and ADA2 was modulated by miR-146-3p in diabetic retinopathy (DR). Actually, pharmacological treatments have been utilized in the potential treatment of ophthalmic diseases. Antagonists of P2X7 receptors prevented ATP-induced neuronal apoptosis in glaucoma, DR, and age-related macular degeneration (AMD); the A_1_ receptor agonists lowered intraocular pressure in glaucoma; the A_2A_ receptor agonists or antagonists reduced neuroinflammation in glaucoma, DR, and AMD; the A_3_ receptor agonists protected retinal ganglion cells (RGCs) from apoptosis in glaucoma [96]. Since the A_1_, A_2A_, and A_3_ receptor were concerned in ophthalmic diseases, and it should be taken into consideration that whether this purinergic signalling was modulated by miRNAs or not.

## Purinergic signalling modulated by miRNAs in musculoskeletal diseases

The P2X7 receptor is the only purinergic signalling found being modulated by miRNAs in osteoarthritis (OA) and intervertebral disc degeneration (IDD). In patients with OA, miR-373 was found decreased [97, 98]. miR-373 inhibited chondrocyte proliferation by suppressing the expression of P2X7 receptors, as well as inflammatory factors such as IL-6 and IL-8, which were regulated by P2X7 receptors [97]. Another study revealed that miR-373 could suppress the inflammation by directly targeting the P2X7 receptor and regulating its expression. In this process, miR-373 could increase by the supplementation of adipose-derived stem cells (ADSCs) [98]. While in IDD, P2X7 receptors were found to play a regulatory role in intervertebral disc degeneration by activating NF-κB signal pathway and thus being implicated in inflammatory responses in the intervertebral disc and the apoptosis of nucleus pulposus cells [99–101]. A later study found that the NF-κB signal pathway could be inhibited by miR-150 through targeting the P2X7 receptor in intervertebral disc degeneration [102]. In this way, miR-150 alleviates the degeneration of the intervertebral disc partially since the IL-1β-induced matrix catabolism, inflammatory response, and apoptosis of the nucleus pulposus cells were inhibited [102].

## Purinergic signalling modulated by miRNAs in other diseases

In other inflammatory diseases, A_2A_ receptors and CD39^+^ Tregs were correlated with miRNAs. The A_2A_ receptor, one of the most important physiological regulators of the inflammatory response, was expressed in almost all organ systems, with the highest abundance in immune cells [103–105]. Compared with the well-known anti-inflammatory role of A_2A_ receptors, miR-214 promotes the release of inflammatory cytokines TNF-α and IL-6 in bone marrow-derived macrophages (BMDMs) [106]. An intriguing fact is that a mutual suppression feedback loop between A_2A_ receptors and miR-214 in inflammation was uncovered, and that explains why the combination of miR-214 antagonist and the A_2A_ receptor agonist exerted more anti-inflammatory effect than using one of them alone [106]. In another study, together with miR-214, miR-15 and miR-16 were revealed to control A_2A_ receptors expression in polymorphonuclear leukocytes (PMNs) via bioinformatic analyses and reporter gene assays [107]. Based on these findings, we could know that the determination of miRNA expression levels might help identify patients with an increased risk of server inflammation, and suppressing A_2A_ receptors and specific miRNAs, including miR-214, miR-15, and miR-16, might help in diminishing inflammation. In sepsis patients, an increased ratio of CD39^+^ Tregs was observed, and the percentage of CD39^+^ Tregs was correlated with the prognosis of patients to the extent [108]. So did miR-155, sepsis patients exhibited a significantly elevated miR-155 level compared to healthy control [109]. miR-155 played a key role in fine-tuning the regulation of lymphocyte subsets, including dendritic cells (DCs), macrophages, B cells, and CD8^+^ and CD4^+^ T cells [110]. Unlike other modulation between purinergic signalling and miRNAs, miR-155 was positively correlated with CD39^+^ Tregs [109]. Therefore, a higher level of miR-155 indicated a more severe conditional and poor prognosis in sepsis patients.

The regulation of miRNA to purinergic signalling has been noticed both in physiological and pathological processes of the lung. Physiologically, miR-150 was reported to reduce P2X7-mediated surfactant secretion of the alveolar epithelial type II cells (AEC II), which cover about 95% of the alveolar surface area [111]. Pathologically, miR-155 could partly alleviate allergic airway inflammation (AAI) by targeting ecto-nucleoside triphosphate diphosphohydrolase1 (ENTPD1) and ENTPD3 (isoforms of ENTPDase/CD39 family) and thus influence ATP homeostasis and activation of DCs [112, 113]. Besides, in ischemic left lungs, both the A_2B_ receptor mRNA and protein concentration showed a significant increase relative to their control right lung counterparts, while miR-27a, which was reported to modulate A_2B_ receptors, was decreased [114].

Dicer is an enzyme essential for miRNA processing. The importance of Dicer in the regulation between miRNAs and purinergic signalling has been described. Though not confirmed, it was revealed that loss of Dicer may impair the expression of miRNAs that are capable of targeting P2X mRNAs in overactive bladder (OAB). Specifically, miR-34a could potentially target P2X1 receptors, and miR-25 could potentially target P2X3 receptors [115].

## Conclusion

Knowing that miRNAs and purinergic signalling have both been seen as potential therapeutic targets, and miRNA could modulate purinergic signalling, the correlation between them is worthy of deeper understanding. Though not all the modulation was validated by directly targeting that being verified via luciferase reporter assay, we can conclude that miRNAs are crucial modulators in purinergic signalling involved diseases according to the present studies (Table 1).

Purinergic signalling was shown to be modulated by miRNAs in several main types of diseases, including cancers, gastrointestinal diseases, circulatory diseases, neurological diseases, ophthalmic diseases, and musculoskeletal diseases. Besides, other diseases, such as inflammation, lung diseases, and overactive bladder, were also involved. Among these diseases, the modulation of miRNAs to purinergic signalling seemed to be relatively more thoroughly studied in cancers, neurological diseases, and gastrointestinal diseases (Table 1). Because, within the 35 scholarly papers which revealed the modulation, 11 of them are concerning cancers, 6 of them concerning neurological diseases, 5 of them concerning gastrointestinal diseases. Intriguingly, the importance of CD73 in the modulation of miRNAs to purinergic signalling was only reflected in cancers until now. miR-150 was reported in 4 different scholarly papers to modulate P2X7 receptors in breast cancer, IDD, MI, and cervical cancer, respectively. The potential of miR-150 shall be noticed.

Due to the regulatory mechanism of miRNAs, a single miRNA can interact with hundreds of mRNA molecules, and reversely, a specific mRNA molecule may be the target of multiple miRNAs. And it was with no surprise embodied in the modulation of miRNAs to purinergic signalling in diseases. miR-187 could modulate the P2X7 receptor and CD73 (Fig. 1). On the other hand, A_2A_ receptors, A_2B_ receptors, P2X7 receptors, and CD73 were reported to be modulated by multiple miRNAs. Apparently, P2X7 receptors and CD73 are modulated by the most significant number of miRNAs than others (Table 1). It was implied that they might be important agents if miRNAs were utilized in future therapeutic strategies.

Beyond the modulation of miRNAs to purinergic signalling, some purinergic signalling modulated or influenced miRNAs in turn. And thus, even some “mutual modulations” were accomplished (Fig. 1). This kind of correlation was shown between miR-214 and A_2A_ receptors, miR-363 and P2X4 receptors, mir-21 and P2X7 receptors, miR-22 and P2X7 receptors, and miR-125b and P2X7 receptors. The interaction makes the potential application of purinergic signalling and miRNAs even more complex and interesting.

## Data Availability

The datasets generated during and/or analyzed during the current study are available from the corresponding author on reasonable request.
